# 508. Title Favipiravir for the Treatment of Coronavirus Disease 2019; A Propensity Score Matched Cohort Study

**DOI:** 10.1093/ofid/ofab466.707

**Published:** 2021-12-04

**Authors:** Rand A Alattar, Shiema A Ahmed, Tasneem Abdallah, Rashid Kazman, Aseelah N Qadmour, Tawheeda Ibrahim, Bassem Alhariri, Shahd H Shaar, Abeer Bajwa, Abeir Alimam, Rabia Qazi, Fatma Ben Abid, Joanne Daghfal, Ali M Eldeeb, Kinda Shukri, Ahmed Elsayed, Fatima Rustom, Musaed S AlSamawi, Alaaeldin A Abdelmajid, Miguel Basulto, Armando Cobian, Mohammed Abu Khattab, Muna Almaslamani, Abdullatif Al Khal, Ali S Omrani

**Affiliations:** 1 Hamad Medical Corporation, Doha, Ad Dawhah, Qatar; 2 CDC- Hamad medical corporation, Doha, Ad Dawhah, Qatar; 3 Hamad medical corporation, Doha, Ad Dawhah, Qatar; 4 Hamad Medical corporation, Doha, Ad Dawhah, Qatar; 5 Hamad bin Khalifa university, Doha, Ad Dawhah, Qatar; 6 HMC, Doha, Ad Dawhah, Qatar; 7 Hamad medical corporation (HMC), Doha, Ar Rayyan, Qatar; 8 Communicable Disease Center, Doha, Ad Dawhah, Qatar

## Abstract

**Background:**

We investigated clinical outcomes of favipiravir in patients with COVID-19 pneumonia.

**Methods:**

Patients who between 23 May 2020 and 18 July 2020 received ≥24 hours of favipiravir were assigned to the favipiravir group, while those who did not formed the non-favipiravir group. The primary outcome was 28-day clinical improvement, defined as two-category improvement from baseline on an 8-point ordinal scale. Propensity scores (PS) for favipiravir therapy were used for 1:1 matching. Cox regression was used to examine associations with the primary endpoint.

**Results:**

The unmatched cohort included 1,493 patients, of which 51.7% were in the favipiravir group, and 48.3% were not receiving supplemental oxygen at baseline (table 1). Favipiravir was started within a median of 5 days from symptoms onset. Significant baseline differences between the two unmatched groups existed, but not between the PSmatched groups (N = 774) (table 1). After PS-matching, there were no significant differences between the two groups in the proportion with 28-day clinical improvement (93.3% versus 92.8%, P 0.780), or 28-day all-cause mortality (2.1% versus 3.1%, P 0.360) (Table 2). Favipiravir was associated with more viral clearance by day 28 (79.8% versus 64.1%, P < 0.001) (table 2). In the adjusted Cox proportional hazards model, favipiravir therapy was not associated 28-day clinical improvement (adjusted hazard ratio 0.978, 95% confidence interval 0.862 –1.109, P 0.726) (Table 3).

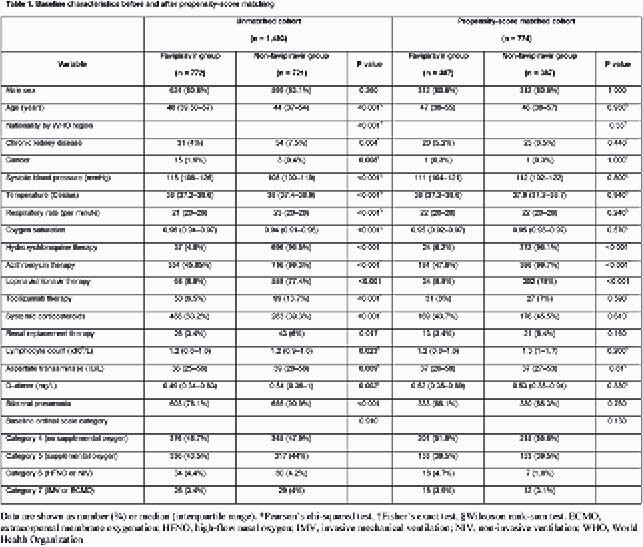

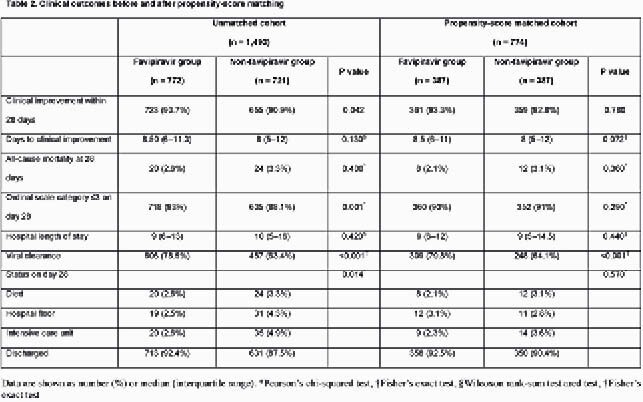

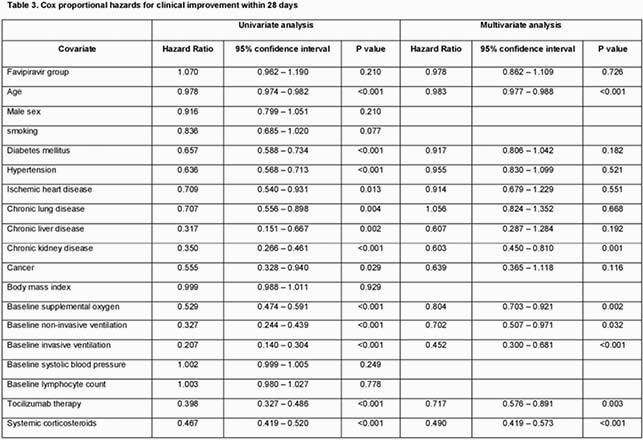

**Conclusion:**

Favipiravir therapy for COVID-19 pneumonia is well tolerated but is not associated with an increased likelihood of clinical improvement or reduced all-cause mortality by 28 days.

**Disclosures:**

**All Authors**: No reported disclosures

